# The Internet Intervention Patient Adherence Scale for Guided Internet-Delivered Behavioral Interventions: Development and Psychometric Evaluation

**DOI:** 10.2196/13602

**Published:** 2019-10-01

**Authors:** Fabian Lenhard, Kajsa Mitsell, Maral Jolstedt, Sarah Vigerland, Tove Wahlund, Martina Nord, Johan Bjureberg, Hanna Sahlin, Per Andrén, Kristina Aspvall, Karin Melin, David Mataix-Cols, Eva Serlachius, Jens Högström

**Affiliations:** 1 Centre for Psychiatry Research Department of Clinical Neuroscience Karolinska Institutet, & Stockholm Health Care Services, Stockholm County Council Stockholm Sweden; 2 Stockholm Health Care Services, Stockholm County Council Stockholm Sweden; 3 Institute of Neuroscience and Physiology, The Sahlgrenska Academy, University of Gothenburg Gothenburg Sweden

**Keywords:** patient compliance, eHealth, measure, internet, cognitive behavioral therapy

## Abstract

**Background:**

Patient adherence is defined as the extent to which a patient complies with medical or health advice. At present, there is a lack of reliable and valid measures specifically designed to measure adherence to internet-delivered behavioral interventions.

**Objective:**

The objective of this study was to develop and psychometrically evaluate a novel measure of adherence to guided internet-delivered behavioral interventions.

**Methods:**

In collaboration with experienced clinicians and researchers in the field, a 5-item, clinician-rated internet intervention Patient Adherence Scale (iiPAS) was developed. The initial scale was tested in a sample of children and adolescents (N=50) participating in internet-delivered cognitive behavioral therapy (ICBT) studies. A revised version of the iiPAS was then administered to a larger sample of children and adolescents (N=148) with various behavioral problems participating in ICBT trials. The scale was evaluated according to a classical test theory framework.

**Results:**

The iiPAS demonstrated excellent internal consistency. Factor analyses revealed one underlying factor, explaining about 80% of the variance, suggesting that the scale captures a homogeneous adherence construct. The iiPAS was strongly associated with objective measures of patient activity in ICBT (number of logins, number of written characters, and completed modules). Furthermore, mid- and posttreatment ratings of the iiPAS were significantly correlated with treatment outcomes. By contrast, objective measures of patient activity in the Web-based platform did not correlate with treatment outcomes.

**Conclusions:**

The iiPAS could be a useful tool to measure adherence in a broad range of internet-delivered behavioral interventions.

## Introduction

### Background

Patient adherence, sometimes also termed compliance, is most broadly defined as “the extent to which the patient’s behavior (in terms of taking medications, following diets, or executing other lifestyle changes) coincides with medical or health advice” [1]. Within the field of psychotherapy, adherence is usually conceptualized within the therapeutic tradition or according to the target condition that is being studied [2]. Accordingly, adherence is measured in various ways, for example, as qualitative or quantitative aspects of homework compliance [3], in-session engagement [4], or patient-therapist alliance [5]. Absence of patient adherence, on the other hand, is often referred to as resistance or ambivalence [6,7], and there are various therapeutic strategies that are specifically designed to target such resistance or ambivalence, such as motivational interviewing [8].

One important aspect of patient adherence is the logical assumption that adherent patients will have better treatment outcomes than nonadherent patients, though the evidence has not always supported this assumption. There is, for example, evidence that adherence to homework assignments in cognitive behavioral therapy (CBT) is associated with better outcomes, with a small to large effect of homework quality and quantity, but with substantial heterogeneity between studies [3,9]. Similarly, adherence to exposure and response prevention homework has been shown to be a strong predictor of short- and long-term outcome in CBT for obsessive-compulsive disorder (OCD) [10,11]. However, results from the field of pediatric anxiety have failed to establish a clear relationship between homework adherence and treatment outcome [12]. For example, in 1 study, neither the number of attended CBT sessions nor completed homework assignments predicted treatment outcome but a 1-item clinician-rated adherence measure did [13]. One study of CBT for anxiety disorders applied a combined measure of adherence, consisting of a weekly clinician rating of homework completion and a rating of engagement in the therapy session. The authors found that a combination of high treatment dose (high session attendance and completed exposure exercises) and high engagement predicted better treatment outcomes [14]. Importantly, it has been suggested that the adherence-outcome relationship could be affected by the quality of the adherence measure, and thus, reliable and valid measures are essential as research and clinical tools [15].

### Internet-Delivered Interventions and Adherence

Internet-delivered behavioral interventions have emerged as an interdisciplinary development of psychotherapy and modern information technology. Internet-delivered behavioral interventions share common features as they mirror face-to-face psychotherapy regarding content and therapeutic techniques but are presented online via a personal login to a website or internet portal. The format usually resembles an online course, with instructive texts, pictures, videos, and written exercises, with the content structured similar to a book with chapters or modules. When therapist contact and feedback are involved, the treatment is referred to as guided or clinician-supported and otherwise as unguided or self-help [16]. Internet-delivered CBT (ICBT) has been one of the most prominent formats of this novel branch of interventions, with over 100 randomized controlled trials (RCTs) conducted in adults [17] and currently 19 RCTs in children and adolescents [18].

Adherence may be particularly difficult to measure in internet-delivered behavioral interventions [19]. In face-to-face treatment, many of the proposed active treatment components, such as behavioral or cognitive strategies, are conducted in-session together with a clinician. In internet-delivered behavioral interventions, the key therapy components are delivered online and practiced without the physical presence of the clinician. Depending on the intervention, the engagement in the active treatment components of the intervention may have low correlation with the number of completed chapters. For instance, patients may go on practicing their homework assignments without necessarily needing to log into the system periodically. Therefore, the number of completed chapters in internet-delivered behavioral interventions is not comparable with the number of completed sessions in face-to-face interventions and, thus, may not be a reliable measure of treatment adherence. As pointed out by Sieverink et al [20], the concept of “intended usage” could be a helpful extension of the traditional definition of adherence. Intended usage is defined as “the extent to which individuals  *should* experience the content (of the intervention) to derive maximum benefit from the intervention, as defined or implied by its creators” [21].

Thus, defining and measuring the core therapeutic concepts and minimal effective use of those strategies may be a vital aspect of any measure of adherence to internet-delivered interventions. However, less than half of the studies in the field apply such a definition, and only few provide a justifiable definition of minimal required usage [20]. Currently, the most prevalent operationalizations of adherence are number of treatment modules completed by patients and number of logins [20,22]. The relationship between adherence and clinical outcome has thus far been limited by inconsistent reporting of adherence and a lack of agreement on measures [18,22].

In summary, there is an important research gap and a need for reliable and valid measures of patient adherence to internet-delivered interventions. We take the perspective that a useful measure of adherence to internet-delivered behavioral interventions should (1) not only capture the structural aspects of the internet format (such as completed chapters or modules) but also consider the intended usage of its central therapeutic aspects (such as use of behavioral strategies) and (2) include relevant, observable behaviors that represent the patients’ adherence in a way that guides clinically meaningful decisions. Thus, the aim of this project was to construct and validate an adherence measure, evaluate its psychometric properties, and establish whether this new measure correlates better with treatment outcome than other traditional measures of adherence (eg, number of logins and completed chapters).

## Methods

### Scale Construction

A novel, clinician-rated measure called internet intervention Patient Adherence Scale (iiPAS) was constructed in a 4-step process following recommendations from the psychometric literature [23].

#### Step 1: Definition of Construct and Context

The first author (FL) conceptualized the scale and drafted a first version with the aim to measure patient adherence as one homogeneous construct. To ensure feasibility, usability, and precision, the scale was intended to be a short and time-efficient measure and assess patient behaviors that would be observable by clinicians. The context of the scale was defined as a clinician-rated measure to be used within clinician-supported, internet-delivered behavioral interventions. The advantage of a clinician-rated scale, compared with a self-rated measure, was justified by the ability of the clinician to assess the adherence of the patient relative to the intended, optimal use of the intervention, which was assumed crucial for the reliability of the instrument [20]. Building on the available evidence presented in the introduction, the scale should include items that capture structural aspects of internet-delivered interventions, equivalent to number of completed chapters, as well as content-related aspects, equivalent to the patients’ engagement in the therapeutic content of the intervention *.*

#### Step 2: Response Format and Initial Item Formulation

To ensure optimal content validity, experienced clinicians and researchers with relevant competence (see “Expert Sample”) were involved in choosing the response format and initial item formulation. The initial draft was circulated among the experts in 2 review rounds and edited iteratively by the first author according to suggestions and comments. The preliminary version of the iiPAS included 5 clinician-rated items rated on a 3-point Likert scale, including anchor labels for the end points of the scale (see description under “Measures”).

#### Step 3: Preliminary Data Collection

The preliminary iiPAS was tested in a small sample (“Patient Sample 1”) to provide basic psychometric information as well as to give clinicians the possibility to test the scale and give feedback regarding usability and applicability.

#### Step 4: Preliminary Examination of Psychometric Properties and Scale Revision

On the basis of additional feedback from step 3, the wording of the items was minimally revised for improved clarity. In addition, the 3-point Likert scale was extended to a 5-point scale to increase response variance. A preliminary psychometric analysis was carried out; results are presented below. For the full psychometric evaluation, the revised iiPAS was then administered to participants in several clinical trials of internet-delivered behavioral interventions (“Patient Sample 2”).

### Participants

#### Expert Sample

The expert sample consisted of 8 experienced researchers and clinicians who had been involved in clinical trials of internet-delivered behavioral interventions (coauthors FL, SV, MJ, MN, PA, KA, TW, and JH) within a Swedish research network called the Child Internet Project or Barninternetprojektet (BiP). The experts were involved in the initial scale development.

#### Patient Sample 1

Initial data were collected from 50 patients, of whom 33 (66%) were children participating in a study of ICBT for pediatric anxiety [24], and 17 (34%) were adolescents participating in a study of ICBT for pediatric OCD [25]. Main inclusion criteria were a primary diagnosis of separation anxiety disorder, social anxiety disorder, generalized anxiety disorder, panic disorder, or specific phobia according to *Diagnostic and Statistical Manual* of Mental Disorders, 4th edition (DSM-IV) and a clinical severity score above 3 on the Clinician Severity Rating (CSR) [26] or a primary diagnosis of OCD with a score of 16 or above on Children’s Yale Brown Compulsive Scale [27]. Clinicians scoring the iiPAS were the same licensed clinical psychologists (N=13) who provided online support as a part of the ICBT interventions. For more detailed information, see the original publications of the respective trials [24,25]. The regional ethical review board in Stockholm, Sweden, approved both studies.

#### Patient Sample 2

Patient sample 2 consisted of 148 children and adolescents with various mental disorders, who together with their primary caretakers participated in different clinical trials. Common inclusion criteria within all trials were (1) age within the age interval for the respective trial (see “Interventions”), (2) internet access, (3) absence of suicidality or another severe condition that required immediate attention or higher priority, and (4) the clinical presentation of a mental disorder that was targeted in the specific trial (tic disorder, social anxiety disorder, generalized anxiety disorder, agoraphobia, panic disorder, specific phobia, separation anxiety disorder, OCD, and nonsuicidal self-injury). All conditions were diagnosed by experienced psychologists using semistructured diagnostic interviews based on DSM-5 criteria [28]. The iiPAS was scored by the treating clinicians (N=21), of whom 20 were licensed psychologists and a licensed nurse. For more detailed information, see the original publications of each trial [24,29-32]. The regional ethical review board in Stockholm, Sweden, approved all trials.

### Interventions

All interventions were provided by researchers and clinicians associated to the BiP network, a collaboration between the Karolinska Institutet and the Child and Adolescent Mental Health Service in Stockholm, with a main research focus on development and evaluation of internet-delivered interventions for childhood mental disorders. All interventions in the current evaluation were clinician-guided ICBT interventions that also included parent support. Clinician advice, support, and feedback were given predominantly via written messages within the secure internet platform or via occasional telephone calls. For a short summary of the interventions, see Table 1. More detailed descriptions can be found in the original publications [29-33].

**Table 1:** Summary of the included internet-delivered cognitive behavior therapy interventions.

Intervention	Target age (years)	Target condition	Number of modules/weeks	Main features
BiP^a^ TIC [29]	7-17	Tourette syndrome/chronic tic disorder	10/10	Habit reversal training or exposure with response prevention
BiP SoFT^c^ [30]	13-17	Social anxiety disorder	9/12	Exposure training and cognitive strategies
BiP OCD^b^ [25,31]	7-17	OCD	12/12	Exposure with response prevention and parental coping strategies
BiP Anxiety [33]	8-12	Mixed anxiety disorders	12/12	Exposure training and child coping strategies
BiP ERITA^d^[32]	13-17	Self-harm	11/12	Emotion regulation strategies and adaptive parental behaviors

^a^BiP: Barninternetprojektet.

^b^OCD: obsessive-compulsive disorder.

^c^SoFT: Social Fobi Tonåring.

^d^ERITA: Emotion Regulation Individual Therapy for Adolescent.

### Procedure

The included clinical trials applied similar but not identical procedures. A description of the common aspects of the procedures is provided here; for a detailed description, see the respective trial publications. Participants in patient samples 1 and 2 were recruited via clinician- or self-referrals. Selection of eligible participants included both telephone screenings with a clinician and an in-person assessment with the child or adolescent and at least one caregiver. Fulfillment of diagnostic criteria was established by a clinician (most often a clinical psychologist) using the Mini-International Neuropsychiatric Interview child version (MINI Kid [34]) or the Anxiety Disorder Interviews Schedule Child and Parent versions (ADIS C/P [26]). Decision about inclusion or exclusion of participants was made after the in-person assessment, followed by treatment allocation.

The BiP TIC and BiP Anxiety trials had a randomized controlled design [24,29]. In BiP TIC, participants were randomized to either habit reversal training or exposure and response prevention. In BiP Anxiety, participants were randomized to either ICBT or an active control condition. The remaining trials had an open, within-group design.

The clinical outcome measures were assessed at pre- and posttreatment. Activity data from the use of the internet platform (number of logins, written characters, login time duration, and completed treatment chapters) were automatically collected during the treatments. The iiPAS was administered by the clinicians participating in the trials, on 2 separate occasions: halfway through the treatment (iiPAS-mid) and at posttreatment (iiPAS-post).

### Measures

#### Internet Intervention Patient Adherence Scale

The iiPAS is a 5-item, clinician-rated measure of patient adherence to internet-delivered behavioral interventions. The 5 items are rated by treating clinicians and cover 5 central aspects of adherence: the patient’s work pace, engagement, communication with the clinician, motivation for change, and login frequency. Each item is rated on a 0 to 4 Likert scale. Thus, the iiPAS-total score ranges from 0 to 20, with 0 indicating no adherence and 20 perfect adherence. The items are designed to generically suit different types of internet-delivered behavioral interventions. A prerequisite, however, is that the intervention is guided, that is, clinician-supported, as opposed to unguided or entirely self-help. The scale is constructed to be applicable to children and adolescents as well as adults. In the case where caregivers are involved in the patient’s treatment, the iiPAS is primarily intended to evaluate the patient’s adherence to treatment rather than the parent’s or carer’s involvement. See Multimedia Appendices 1 and 2 for a full copy of iiPAS in Swedish and English and scoring instructions. For permission requests to use or translate the scale, please contact the corresponding author.

#### Clinical Global Impression-Severity

Clinical Global Impression-Severity (CGI-S [35]) is a commonly used clinician-rated measure of global illness severity. Clinical severity is rated on a 7-point Likert scale, ranging from “normal, not ill” to “severely ill.” The CGI-S is validated as a measure of clinical effectiveness within clinical trials as well as within routine clinical practice [36].

#### Clinician Severity Rating

CSR is rated in conjunction with the clinician-administered ADIS [26], which is a semistructured interview and is considered the gold standard in the assessment of pediatric anxiety disorders. The CSR is a clinician rating of clinical severity between 0 and 8, with 0 indicating absence of symptoms (or no disturbance in functioning and/or disability**)**, 8 maximal severity, and 4 the cut-off for clinical severity (ie, meeting diagnostic criteria for the disorder). ADIS has previously shown excellent interrater reliability [37] and test-retest reliability [38].

#### Platform Usage Variables

The internet treatment platform that the patients logged in to for accessing treatment material (texts, videos, and exercises) provided user data on number of logins to the platform, duration of logged in time, and number of completed chapters and written characters by the patients in communication and exercises. The number of completed chapters was expressed in percentage relative to total number of chapters because of the different number of chapters in different interventions.

### Statistical Analyses

#### Reliability Analyses

Cronbach alpha was calculated as a measure of internal consistency. An exploratory factor analysis (EFA) was conducted regarding the factor structure of the iiPAS, including the Kaiser-Meyer-Olkin (KMO) measure of sampling adequacy to determine the adequacy of the data for factor analysis as well as a Bartlett Test of Sphericity to determine the factorability of the five items.

#### Validity Analyses

Pearson correlations were used to explore convergent validity, defined as the association of the iiPAS with platform usage variables. In variables where outliers with large leverage were identified, Spearman correlation coefficient was used. The intercorrelations of iiPAS between the halfway treatment time point (iiPAS-mid) and at the end of treatment (iiPAS-post) were calculated. For some calculations, an iiPAS total score was used, which is the sum of iiPAS-mid and iiPAS-post, to obtain a measure of adherence that represents the whole treatment period from start to end of treatment. Regarding criterion validity, the association between iiPAS and symptom severity change from pre- to posttreatment was calculated. Because the available measures, CSR (in the BiP Anxiety group) and CGI-S (in the remaining groups), are similar clinician-rated, 1-item Likert scales, but not identical, the CSR and CGI-S change scores were z-transformed and then combined into a single new variable (“symptom severity change”), which was then further used in the analyses. There were only a few missing values in the dataset (2.7%), and missingness was, therefore, not further handled but listwise excluded from the respective analyses. Analyses were conducted in SPSS Statistics version 24 (IBM Corp) and R (R Core Group [39]).

## Results

### Study 1: Initial Evaluation

An initial version of the iiPAS was administered to 50 children and adolescents, aged between 7 and 17 years, receiving ICBT for OCD or anxiety disorders. Preliminary analyses of Cronbach alpha indicated excellent internal consistency (alpha=.93).

### Study 2: Full Psychometric Evaluation

#### Sample Characteristics

The majority of the 148 participants were female (n=89, 60%), with a mean age of 12.7 years. Table 2 displays a detailed description of the characteristics of the participants in each trial.

Table 3 presents descriptive statistics for iiPAS at the 2 time points midtreatment and posttreatment and for the summary value, iiPAS-total, as well as platform usage variables.

**Table 2:** Sample characteristics (N=148).

Characteristic		BiP TIC (n=23)	BiP SoFT (n=30)	BiP OCD (n=24)	BiP Anxiety (n=46)	BiP ERITA (n=25)	Total
**Age, years:months**							
	Mean (SD)	12:3 (2.56)	15:0 (1.22)	11:5 (2.75)	9:11 (1.36)	15:9 (1.31)	12:7 (2.94)
	Min-max	8-17	13-17	8-17	8-12	13-17	8-17
**Sex,** **n (%)**							
	Female	8 (35)	25 (83)	14 (58)	23 (50)	19 (76)	89 (60.1)
	Male	15 (65)	5 (17)	10 (42)	23 (50)	1 (4)	55 (37.1)
	Other/nonbinary sex	N/A^a^	N/A	N/A	N/A	5 (20)	4 (2.7)
**Principal diagnosis, n (%)**							
	Tourette syndrome/chronic tic disorder	23 (100)	—^b^	—	—	—	23 (15.5)
	Social anxiety disorder	—	30 (100)	—	5 (11)	—	35 (23.6)
	OCD^c^	—	—	24 (100)	—	—	24 (16.2)
	Separation anxiety disorder	—	—	—	17 (37)	—	17 (11.4)
	Generalized anxiety disorder	—	—	—	12 (26)	—	12 (8.1)
	Specific phobia	—	—	—	9 (20)	—	9 (6.0)
	Panic disorder	—	—	—	3 (7)	—	3 (2.0)
	Nonsuicidal self-injury	—	—	—	—	25 (100)	25 (16.8)

^a^N/A: not applicable.

^b^No occurrence.

^c^OCD: obsessive-compulsive disorder.

**Table 3:** Descriptive statistics for internet intervention Patient Adherence Scale and platform usage variables.

Variable	Mean (SD)	Total score percentiles				
		5th	25th	50th	75th	95th
iiPAS^a^-mid	13.43 (4.98)	5	10	15	17	20
iiPAS-post	11.27 (6.01)	0	7	12	16	19
iiPAS-total	24.74 (10.39)	7	17	26	34	39
Number of logins	32 (16.49)	—^b^	—	—	—	—
Logged in time (min)	2277^c^ (5673.92)	—	—	—	—	—
Number of written characters	18,135^d^ (50,624.17)	—	—	—	—	—
Completed chapters (%)	74.4 (25.8)	—	—	—	—	—

^a^iiPAS: internet intervention Patient Adherence Scale.

^b^Percentiles not calculated.

^c^Median=762.

^d^Median=5510.

#### Internal Consistency

Table 4 displays Cronbach alpha for iiPAS-mid and iiPAS-post across the included clinical studies. The internal consistency of the iiPAS was excellent at midtreatment as well as at posttreatment.

**Table 4:** Cronbach alpha for internet intervention Patient Adherence Scale.

Study	iiPAS^a^-mid	iiPAS-post
BiP^b^ TIC	.90	.95
BiP SoFT^c^	.96	.96
BiP OCD^d^	.91	.92
BiP Anxiety	.96	.94
BiP ERITA^e^	.94	.97
All trials combined	.93	.95

^a^iiPAS: internet intervention Patient Adherence Scale.

^b^BiP*:* Barninternetprojektet

^c^SoFT*:* Social Fobi Tonåring

^d^OCD*:* Obsessive Compulsive Disorder

^e^ERITA*:* Emotion Regulation Individual Therapy for Adolescents

#### Factor Structure

For iiPAS-mid, a KMO=0.89 and Bartlett Test of Sphericity χ^2^_10_=578.5, *P*<.001 indicated factorability. The results of the EFA indicated a single factor with an eigenvalue of 3.97 that explained 79.3% of the variance. For iiPAS-post, KMO was 0.85 and Bartlett Test of Sphericity χ^2^_10_=787.0, *P*=.001, again indicating factorability. The EFA resulted in a single factor with an eigenvalue of 4.18 that explained 83.6% of the variance. Multimedia Appendix 3 presents communalities and factor loadings in both EFAs.

#### Convergent Validity

iiPAS-mid, iiPAS-post, and iiPAS-total scores were positively and significantly correlated with platform usage variables (*r*=0.25-0.79), apart from nonsignificant correlations between iiPAS and logged in time. There was also a substantial and significant correlation between iiPAS-mid and iiPAS-post (*r*=0.78). Visual inspection of the scatterplots did not indicate any nonlinear associations. Table 5 presents convergent validity correlations.

#### Criterion Validity

iiPAS-mid, iiPAS-post, and iiPAS-total scores correlated positively and significantly with the standardized symptom severity change score (*r*=0.17-0.19; Table 5). In contrast, number of logins, logged in time, written characters, and percentage of completed chapters were not significantly correlated with symptom severity change. A post hoc item-per-item analysis of the iiPAS-mid items regarding the symptom severity change score revealed a significant correlation with 2 of the 5 items, item 2 (“engagement with exercises”; *r*=0.21, *P*=.01), and item 4 (“motivation for change”; *r*=0.19, *P*=.02).

**Table 5:** Internet intervention Patient Adherence Scale correlations (*P* values) with platform usage variables and symptom severity change.

Measure	iiPAS^a^-mid (*P* value)	iiPAS-post (*P* value)	iiPAS-total (*P* value)	Number of logins (*P* value)	Logged in time (*P* value)	Written characters (*P* value)	Completed chapters (*P* value)
iiPAS-post	*.78^b^ (<.001)*	—^c^	—	—	—	—	—
iiPAS-total	*.93 (<.001)*	*.95 (<.001)*	—	—	—	—	—
Number of logins	*.53 (<.001)*	*.57 (<.001)*	*.59 (<.001)*	—	—	—	—
Logged in time	.15 (.07)	.06 (.47)	.11 (.21)	*.19 (.03)*	—	—	—
Written characters	*.25 (.003)*	*.31 (<.001)*	*.31 (<.001)*	*.36 (<.001)*	.10 (.24)	—	—
Completed chapters	*.70 (<.001)*	*.79 (<.001)*	*.79 (<.001)*	*.58 (<.001)*	.09 (.29)	*.26 (<.001)*	—
Symptom severity change	*.17 (.04)*	*.19 (.02)*	*.18 (.04)*	.07 (.40)	−.04 (.61)	−.01 (.94)	.10 (.25)

^a^iiPAS: internet intervention Patient Adherence Scale.

^b^Italicized values indicating significant correlations at the <.05 level.

^c^Not applicable.

## Discussion

### Principal Findings

The objective of this project was to develop and psychometrically evaluate a novel measure of patient adherence in internet-delivered behavioral interventions. The measure was designed in close collaboration with experienced clinicians and researchers within the field and resulted in a clinician-rated, 5-item scale that was tested in a sample of children and adolescents with various behavioral disorders. Descriptive statistics indicated an appropriate range and variability of the iiPAS. The scale demonstrated excellent internal consistency. Consistently, factor analyses showed that the iiPAS measures one underlying factor, in agreement with the theoretical assumption that patient adherence within the context of internet interventions can be understood as one homogeneous construct.

Objective behavioral data were available through automatically registered internet platform data of number of logins, logged in time, written characters, and percentage of completed chapters. The iiPAS was significantly associated with those objective variables, apart from logged in time, which strongly suggests that the iiPAS indeed measures key aspects of patient adherence. The iiPAS was also significantly associated with symptom severity changes after treatment. Post hoc analyses revealed that 2 of the 5 items at midtreatment, engagement and motivation to change, were particularly associated with a decrease in symptoms. The midtreatment administration of the iiPAS may be especially interesting from a clinical point of view, as half of the internet treatment is still ahead. For example, a low iiPAS midtreatment score could inform the clinician that the adherence of the patient should be addressed for optimal outcome. The strong correlation of the iiPAS-mid and iiPAS-post suggests that little change in adherence is to be expected in the second half of the treatment. This could mean that other interventions such as motivational interventions might be needed to increase adherence, or that the intervention might need to be intensified or augmented with telephone, video calls or in-person therapist support.

Interestingly, none of the objective platform usage variables were associated with symptom severity change at posttreatment. This could possibly indicate that the mere adherence to the formal structure of an internet intervention (logging in, writing text, and completing chapters) is less important for the clinical outcome than actively engaging with the therapeutic content that is presented in the intervention (such as testing new behavioral or cognitive strategies and delivering homework exercises with high quality). The existing literature on patient adherence supports that quality of homework exercise completion is a better predictor of treatment outcome than number of completed exercises [10,13]. However, studies that are specifically designed to clarify the importance of adhering to the formal structure of a treatment in comparison with adhering to the therapeutic content are needed.

### Limitations and Future Directions

Our analyses included diverse conditions, ranging from anxiety disorders, OCD, tics/Tourette’s disorder, and self-harming behaviors. Future studies should aim for an even broader evaluation of the iiPAS in additional diagnoses and symptoms that are currently treated with internet interventions, especially in the behavioral medicine field, where about half of the internet treatment trials are conducted [18]. Moreover, although the scale was designed to be used across the life span, the age range of the participants is limited. Further validation work in adult samples is warranted. In addition, the iiPAS was evaluated in clinical trials in Sweden and, albeit, with projects from different research groups, all were located at the same university and used the same internet platform, limiting the generalizability to other contexts. Future studies should evaluate the iiPAS in other languages as well as in different cultural, health care, and academic environments.

Importantly, the iiPAS was designed to be useful in a wide range of internet-delivered, clinician-supported internet interventions of different therapeutic schools. However, the interventions in this study are all based on a CBT framework. An important next step would be to test the iiPAS in different internet-delivered behavioral interventions. Regarding the reliability analyses, an important aspect that was omitted in this study was the calculation of interrater reliability. Due to the scoring format of the iiPAS, this would require 2 clinicians for each patient, which was not feasible at the current stage of evaluation but would be important to take into account in future studies.

Finally, the sample size in this study was big enough for a global evaluation but too small to conduct detailed separate analyses for each specific condition or age group. A larger sample would allow analysis of the association between patient adherence and clinical outcomes in greater detail with regards to specific patient characteristics such as age, comorbidity, and symptom severity at baseline.

### Conclusions

To summarize, the iiPAS demonstrated sound psychometric characteristics in a clinical sample of children and adolescents with a broad range of psychiatric and behavioral problems. The iiPAS appears to be a valuable tool to measure and study different aspects of adherence within the context of clinician-guided, internet-delivered behavioral interventions. Further evaluations within different age groups, types of internet-delivered interventions, and patient populations are warranted.

## Appendix

Multimedia Appendix 1 Internet intervention Patient Adherence Scale—Swedish.

Multimedia Appendix 2 Internet intervention Patient Adherence Scale—English.

Multimedia Appendix 3 Supplemental tables—factor analyses: communalities and factor loadings.

## Abbreviations

ADIS: Anxiety Disorders Interview Schedule
CBT: cognitive behavioral therapy
CGI-S: Clinical Global Impression-Severity
CSR: Clinician Severity Rating
DSM: Diagnostic and Statistical Manual of Mental Disorders
EFA: exploratory factor analysis
ICBT: internet-delivered cognitive behavioral therapy
iiPAS: internet intervention Patient Adherence Scale
KMO: Kaiser-Meyer-Olkin
OCD: obsessive-compulsive disorder
RCT: randomized controlled trial
