# Supplementary material for: The Internet Intervention Patient Adherence Scale for Guided Internet-Delivered Behavioral Interventions: Development and Psychometric Evaluation
Source: J Med Internet Res. 2019 Oct 1;21(10):e13602. doi: 10.2196/13602 (PMC6774571; doi:10.2196/13602)
Supplement: Multimedia Appendix 1 [file jmir_v21i10e13602_app1.pdf]

iiPAS
internet intervention Patient Adherence Scale

Patient/ID: ______________________

Bedömare: ______________________

Datum: ______________________

Halvtidsskattning (skatta start – halvtid) ☐

Avslutningsskattning (skatta halvtid – avslut) ☐

1) Arbetstempo: ligger patienten i fas med behandlingen (t.ex. jobbar med kapitel 6 under vecka 6)?

0	1	2	3	4
jobbar ej med behandlingen, inaktiv				är helt i fas med behandlingen

2) Engagemang i övningar: I vilken utsträckning lägger patienten ner tid och engagemang på de övningar som presenteras i behandlingen?

0	1	2	3	4
har ej gjort några övningar				gjort alla övningar, svarat med intresse och engagemang

3) Kommunikation med behandlaren: I vilken utsträckning engagerar sig patienten i kommunikation med behandlaren, svarar på meddelanden och tar själv initiativ till diskussionsämnen och/eller frågor?

0	1	2	3	4
svarar ej på meddelanden eller frågor				pågående dialog med behandlaren, initierar själv kommunikation

4) Förändringsmotivation: I vilken utsträckning är patienten villig att aktivt testa och använda de strategier som presenteras i behandlingen (t.ex. beteende- eller kognitiva interventioner)?

0	1	2	3	4
använder sig inte av de presenterade strategierna				jobbar aktivt och regelbundet med de presenterade strategierna

5) Inloggningsfrekvens: Hur ofta är patienten aktiv i internetbehandlingen?

0	1	2	3	4
är inte aktiv				ofta

© 2019 - För förfrågningar om att använda, modifiera eller översätta skalan kontakta Fabian Lenhard, PhD, Fabian.lenhard@ki.se.

iiPAS
Instruktion

iiPAS skattas på barnets, ungdomens eller den vuxne patientens aktivitet i internetbehandlingen. Syftet med iiPAS är att mäta patientens följsamhet (adherens) i internetbehandlingar av olika slag.

Skattningarna avser perioden från behandlingens början till halvtid (t.ex. slutet av vecka 6 i en 12 veckor lång behandling) och från halvtid till avslut (i samma exempel vecka 7 till 12). Annan skattningsfrekvens är möjlig (t.ex. veckovis), men halvtids- och avslutningsskattning har visat sig ge pålitlig och kliniskt användbar information.

I en behandling med föräldradel eller där barn och föräldrar arbetar gemensamt med behandlingen (och där barnets adherens är i stora delar beroende av förälderns engagemang) skattas skalan med fokus på barnets aktivitet i behandlingen (inte huruvida föräldern stöttar barnet på ett önskvärt sätt). Detta eftersom skalan just ska mäta patientens adherens i behandlingen, oavsett olika bakgrundsfaktorer som möjliggör en viss grad av adherens för patienten. Ifall det är av intresse kan en separat föräldraskattning göras med iiPAS.

Ytterligheterna i skalstegen kan i vissa fall behöva anpassas till hur olika internetbehandlingar är upplagda. Exempelvis avseende item 5, inloggningsfrekvens: behandling A kan vara upplagt på ett sådant sätt att det är idealiskt om patienten loggar in 4 – 5 gånger i veckan. Behandling B däremot är gjort på ett sådant sätt att patienten följer upplägget optimalt om han/hon loggar in 1 gång i veckan. Det vill säga, såväl inloggningar 5 gånger i veckan som inloggningar 1 gång i veckan kan skattas som en 4:a, utifrån det specifika behandlingsupplägg i behandling A respektive B. En 4:a på skalan behöver därför tolkas utifrån den aktuella behandlingen, men ska alltid stå för det mest önskvärda adherensbeteendet från patientens sida på respektive item.

För att uppnå god inter-rater reliabilitet behöver därför bedömare inom en behandling komma överens om hur skalstegen ska skattas, samt att ytterligheterna (4:orna) behöver definieras (fundera på frågan ”Vilka adherensbeteenden skulle vi se hos en patient som följer behandlingsupplägget på ett helt idealiskt sätt?”).

© 2019 - För förfrågningar om att använda, modifiera eller översätta skalan kontakta Fabian Lenhard, PhD, Fabian.lenhard@ki.se.
