# Supplementary material for: The Internet Intervention Patient Adherence Scale for Guided Internet-Delivered Behavioral Interventions: Development and Psychometric Evaluation
Source: J Med Internet Res. 2019 Oct 1;21(10):e13602. doi: 10.2196/13602 (PMC6774571; doi:10.2196/13602)
Supplement: Multimedia Appendix 2 [file jmir_v21i10e13602_app2.pdf]

iiPAS

internet intervention Patient Adherence Scale

Patient/ID: ______________________

Assessor: ______________________

Date: ______________________

Mid-treatment assessment ☐
(period from start of treatment – mid-treatment)

End of treatment assessment ☐
(period from mid- to end of treatment)

1) Pace of work: is the patient in phase with the treatment (e.g. working on chapter 6 during week 6)?

0	1	2	3	4
Is not working on the treatment, inactive				Is completely in phase with the treatment

2) Involvement in exercises: To what extent does the patient invest time and is actively involved in the exercises that are presented in the treatment?

0	1	2	3	4
Has not done any exercises				Has done all exercises, responded with interest and involvement

3) Communication with the treatment provider: To what extent is the patient involved in communication with the treatment provider, responds to messages and takes the initiative in topics of discussion and/or asking questions?

0	1	2	3	4
Does not respond to messages or questions				Ongoing dialogue with the treatment provider, initiates communication

4) Motivation for change: To what extent is the patient willing to actively try and use the strategies presented in the treatment (e.g. behavioural and cognitive interventions)?

0	1	2	3	4
Does not use the presented strategies				Works actively and regularly on the presented strategies

5) Login frequency: How often is the patient active in the internet treatment?

0	1	2	3	4
Is not active				Often

© 2019 - For permission requests to use, modify or translate the scale, contact Fabian Lenhard, PhD, Fabian.lenhard@ki.se.

iiPAS

Instructions

iiPAS assesses the patient's engagement in the internet treatment. It is suitable for children, adolescents and adults. The purpose of iiPAS is to measure the patient's adherence to a broad range of therapist-guided internet-based interventions.

The assessments refer to the period from the start of the treatment to mid-treatment (e.g. the end of week 6 in a 12 week long treatment) and from mid-treatment to the end of treatment. A different assessment frequency is possible (e.g. weekly) but a mid-treatment and an end of treatment assessment should always be made.

For a treatment with a parental part or where children and parents work together on the treatment (and where the child's adherence to a major extent is dependent on the parent's involvement) the scale is assessed with a focus on the child's activity in the treatment (not whether the parent supports the child in a desirable way). This is because the scale should just measure the patient's adherence to the treatment, regardless of various background factors that enable a certain degree of adherence for the patient. If it is of interest, a separate parent assessment can be made with iiPAS.

The extremes of the scale steps may in certain cases need to be adapted to how different internet treatments are set up. For example in relation to item 5, login frequency: treatment A could be set up in such a way that it is ideal that the patient logs in 4 – 5 times a week. Treatment B on the other hand, is done in such a way that the patient follows the set up optimally if he/she logs in once a week. This means that logins 5 times a week as well as logins once a week could be assessed as a 4, based on the specific treatment set up of treatment A and B respectively. A 4 on the scale therefore should always represent the most desirable adherence behaviour from the patient's side for the respective items.

In order to achieve good inter-rater reliability, assessors of a treatment must agree on how the scale steps should be assessed and that the extremes (the 4's) need to be defined (think about the question "Which adherence behaviours would we see in a patient that follows the treatment plan in a way that is completely ideal?").

© 2019 - For permission requests to use, modify or translate the scale, contact Fabian Lenhard, PhD, Fabian.lenhard@ki.se.
