# Supplementary material for: The Internet Intervention Patient Adherence Scale for Guided Internet-Delivered Behavioral Interventions: Development and Psychometric Evaluation
Source: J Med Internet Res. 2019 Oct 1;21(10):e13602. doi: 10.2196/13602 (PMC6774571; doi:10.2196/13602)
Supplement: Multimedia Appendix 3 [file jmir_v21i10e13602_app3.pdf]

Supplement 3

Table S1: Communalities and factor loadings for iiPAS at the halfway time point.

	Communalities	Factor loadings
iiPAS item 1	.70	.84
iiPAS item 2	.80	.89
iiPAS item 3	.77	.88
iiPAS item 4	.67	.82
iiPAS item 5	.79	.89

Table S2: Communalities and factor loadings for iiPAS at the post-treatment time point.

	Communalities	Factor loadings
iiPAS item 1	.78	.88
iiPAS item 2	.88	.94
iiPAS item 3	.81	.90
iiPAS item 4	.65	.81
iiPAS item 5	.87	.93
